# Natural Language Processing Applications for Computer-Aided Diagnosis in Oncology

**DOI:** 10.3390/diagnostics13020286

**Published:** 2023-01-12

**Authors:** Chengtai Li, Yiming Zhang, Ying Weng, Boding Wang, Zhenzhu Li

**Affiliations:** 1 School of Computer Science, Faculty of Science and Engineering, University of Nottingham Ningbo China, Ningbo 315100, China; 2 Hwa Mei Hospital, University of Chinese Academy of Sciences, Ningbo 315010, China

**Keywords:** natural language processing, computer-aided diagnosis, oncology, electronic health records, electronic medical records

## Abstract

In the era of big data, text-based medical data, such as electronic health records (EHR) and electronic medical records (EMR), are growing rapidly. EHR and EMR are collected from patients to record their basic information, lab tests, vital signs, clinical notes, and reports. EHR and EMR contain the helpful information to assist oncologists in computer-aided diagnosis and decision making. However, it is time consuming for doctors to extract the valuable information they need and analyze the information from the EHR and EMR data. Recently, more and more research works have applied natural language processing (NLP) techniques, i.e., rule-based, machine learning-based, and deep learning-based techniques, on the EHR and EMR data for computer-aided diagnosis in oncology. The objective of this review is to narratively review the recent progress in the area of NLP applications for computer-aided diagnosis in oncology. Moreover, we intend to reduce the research gap between artificial intelligence (AI) experts and clinical specialists to design better NLP applications. We originally identified 295 articles from the three electronic databases: PubMed, Google Scholar, and ACL Anthology; then, we removed the duplicated papers and manually screened the irrelevant papers based on the content of the abstract; finally, we included a total of 23 articles after the screening process of the literature review. Furthermore, we provided an in-depth analysis and categorized these studies into seven cancer types: breast cancer, lung cancer, liver cancer, prostate cancer, pancreatic cancer, colorectal cancer, and brain tumors. Additionally, we identified the current limitations of NLP applications on supporting the clinical practices and we suggest some promising future research directions in this paper.

## 1. Introduction

**Figure 1:**
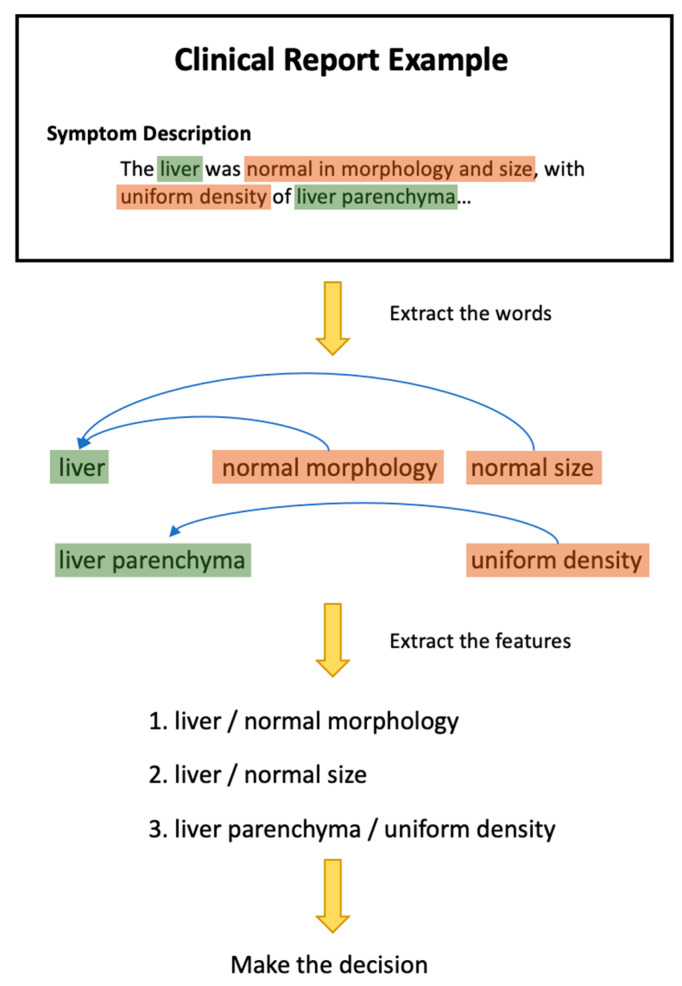
An example of CAD using NLP.

Natural language processing (NLP) is the term used by algorithms to understand the speech or text of humans. The early NLP algorithms were constructed using hard or heuristic rules. With the developments of artificial intelligence (AI), the powerful AI-related technologies have emerged in the NLP field. Some AI methods can even detect patterns or features humans cannot find. In addition, the clinical and research data related to oncology are growing rapidly [1] to meet the data needs of AI algorithms. Electronic Health Records (EHR) or Electronic Medical Records (EMR) provide easy access to the vast amount of patient data collected in clinical practice [2]. Most data in EHR or EMR are recorded in the form of unstructured data (clinical notes and reports), with a small amount recorded in the form of structured data (patient demographics, vital signs, lab tests) [3]. Previously, computer-aided diagnosis (CAD) was mainly based on medical images, which were responsible for highlighting the lesion area to assist the physician in making a diagnosis or making triage on the image, such as using medical images to detect breast cancer [4]. However, the text also contains a lot of critical information for the computer to diagnose. Additionally, the amount of text in a typical clinical report is excessive and using the computer to make a preliminary diagnosis can significantly improve the doctor’s efficiency. In the context of the recent rapid developments of NLP’s internal technical power, external data resources, and practical needs, there is a trend to apply NLP to CAD in oncology. An example is shown in Figure 1. During this research, no secondary data articles were found in literature reviews on NLP applications for CAD in oncology, so it is considered opportune to conduct this literature review. Through this research, it is possible to analyze the articles of primary research data and applications of NLP for CAD in oncology. This multidisciplinary review aims to summarize the current research trends in NLP applications for CAD in oncology and provide guidance for researchers in AI and medicine on designing better NLP applications. Moreover, this paper seeks to answer the three research questions (RQs):

RQ1: What are the current trends of NLP applications for CAD in oncology?
RQ2: What are the limitations and challenges?
RQ3: What are the promising future directions?

We first obtain the required medical data, most of which are EHR or EMR, and the remaining small portion of the medical data is stored in a server in a similar electronic format. For rule-based models, since the rules developed are text-based, we will directly extract text as features and then input them into the model to make the classifications. For traditional-based models, we process the input text as vectors. Next, we manually extract some main feature vectors for the model to learn. Finally, we feed the feature vectors and input vectors that need to be learned into the model for classification. For DL-based models, we convert the text into vectors and enter them directly into the model, allowing for the DL model to learn the high-level features of the data for classification on its own.

We found a certain disconnect between the applications or research of NLP for CAD in oncology and the current theoretical approach to NLP. We believe that this review will lead to a deeper understanding of the whole NLP field and address part of the mismatch between theoretical and practical approaches. Moreover, we analyzed the current approach’s limitations and provided some possible future areas for further research in NLP based on these limitations. In summary, this review has the following main objectives:

- Conclude some AI- and NLP-related concepts and algorithms to help people quickly understand the basics in the field;
- Summarize and analyze the recent decade of research and application of NLP for CAD to various tumors or cancers;
- Provide a more detailed discussion of the current models in the field;
- Identify challenges with the development of NLP in oncology;
- Give some suggestions and directions for the future development of NLP;

The structures of the review are as follows: Section 2 provides the related theoretical foundation, Section 3 introduces the literature’s search method, Section 4 presents and analyses the results of the selected literature from several perspectives, Section 5 summarizes and discusses the current challenges and future trends in the field, and we conclude the review in Section 6.

## 2. Theoretical Foundation

### 2.1. Related NLP Concepts

In terms of the nature of algorithms, NLP approaches can be divided into three categories: rule-based NLP, traditional machine learning (ML)-based NLP, and deep learning (DL)-based NLP [5]. Rule-based NLP was an early approach to NLP, referring to researchers using their own set of hard or heuristic rules for processing text. The performance of rule-based NLP depends on the complexity and generalization capability of the rules. Designing a set of rules requires a significant human investment. Moreover, managing rules can be awfully costly when the number of rules reaches a certain size [6]. Traditional ML-based NLP allows for models to be established from data from a self-learning perspective. Compared to rule-based NLP, traditional ML-based NLP performs better and is easier to model. DL was introduced to NLP after the achievements of ImageNet [7] and Swithchboard [8]. The DL-based NLP approach relies on less human intervention, with the extraction of features depending entirely on the computer itself during the modeling process. Therefore, DL-based NLP is data-driven and performs better than traditional ML-based NLP. However, this feature also causes the poor interpretability of DL-based NLP, which means people cannot understand the kernel of a model.

As for the performance evaluation of NLP models, the commonly used evaluation metrics are shown in Table 1.

### 2.2. Related AI Methods

In traditional ML-based algorithmic models, some classic models are well-known: support vector machine (SVM), decision tree, and logistic regression. SVM can be described as a system that uses a hypothesis space of linear functions in a high-dimensional feature space. It is trained with learning algorithms derived from optimization theory [9]. In contrast to deep neural networks, SVM are adept at situations where the number of feature dimensions is greater than the number of samples. The decision tree is a formalism for learning how to classify by analyzing known instances [10]. In the decision tree, a tree structure with different branches is constructed for the sample features, consisting of directed edges and nodes, with the middle node representing a feature and the final leaf node representing a category or regression value. Logistic regression is a generalized linear classification. The application scenario for logistic regression is where the data can only be classified by a unit step function. Instead of a unit step function, we use the log odds function in logistic regression to make the optimization process differentiable.

We used to use the convolutional neural network (CNN) [11] and recurrent neural network (RNN) [12] as DL models for NLP tasks, but in recent years the Transformer [13] has taken over almost all NLP tasks. CNN extracts semantic information of context using the convolutional method, which is an abstraction of higher-order features. The contextual relationship of NLP data has a strong sequential character, while CNN is justified to handle NLP because its multilayer network structure enables a richer cascaded semantic representation. Moreover, the windows with different sizes of CNN can extract different degrees of semantic features. RNN has the feature that the output of the previous moment is used as the input of the next moment, which is suitable for processing sequential data. However, when the sequence data length is long, it can lead to gradient disappearance or gradient explosion. This is due to the RNN model being affected by the chain rule of derivation in the backpropagation process. Later variants of the RNN model address this problem. Transformer introduces a feed-forward network architecture that completely eliminates the need for convolution and recursion. In contrast to the time-series RNN, Transformer can be processed in parallel. At the core of Transformer is a self-attention mechanism, where each character is able to calculate an attention score against all other characters. This calculation captures long-range dependencies.

### 2.3. NLP Pipelines

**Figure 2:**
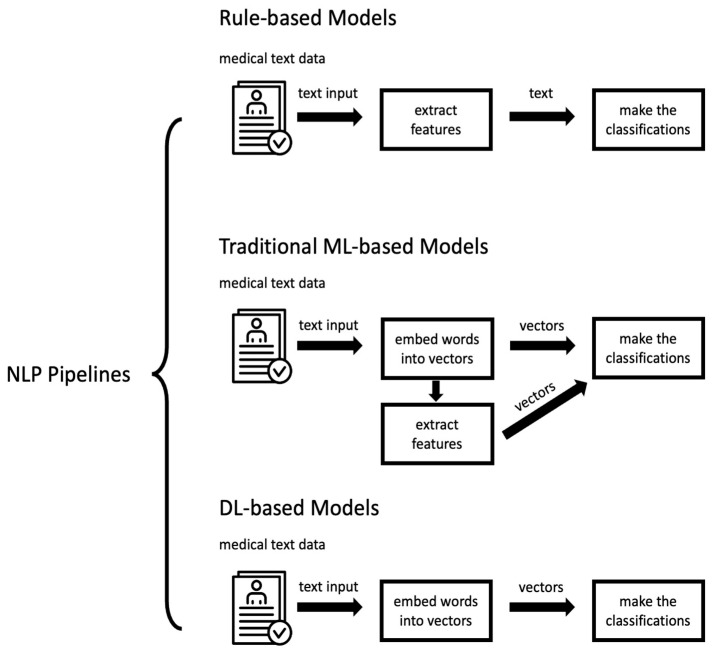
The overview pipeline of NLP applications for CAD in oncology: rule-based models, traditional ML-based models, and DL-based models.

This review focuses on specific research and applications of NLP for CAD in oncology. The details of the entire NLP pipeline will vary slightly depending on the type of core model studied. These differences are mainly reflected in the specific implementation of the extraction feature engineering step and the input of the model. For this purpose, we mapped three pipelines corresponding to three different approaches, as shown in Figure 2, to illustrate the complete pipeline of NLP applications for companion diagnostics. Note that our pipeline diagrams are a general overview of the process, so they cannot represent the precise NLP pipeline in the specific study.

## 3. Materials and Methods

This review produced the literature search for the application of NLP for CAD in oncology using keywords. We searched three electronic databases, PubMed, Google Scholar, and ACL Anthology, for relevant literature between 2012 and 2022. In addition, some search criteria were used to maximize search coverage: **((ALL(“NLP”) OR ALL(“Natural Language Processing”)) AND (ALL(“Cancer”) OR ALL(“Tumor”) OR ALL(“Oncology”)) AND (ALL(“Computer Aided Diagnosis”)))**. The search results were restricted to journals and conferences; books were not included.

**Figure 3:**
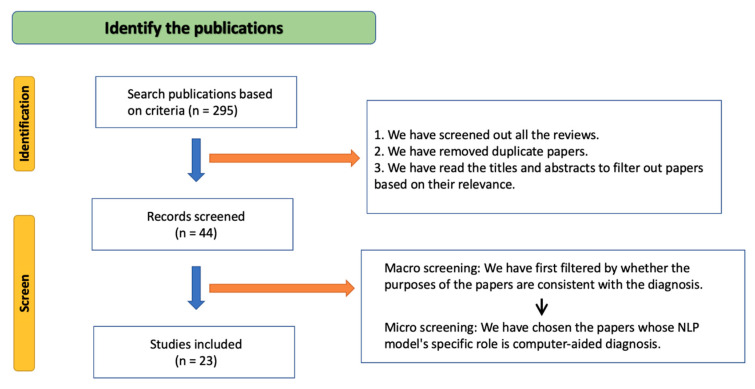
The flowchart of literature identification and screen process.

Based on the search, we first identified 295 relevant publications, including 46 papers from PubMed, 245 papers from Google Scholar, and 4 articles from ACL Anthology. Then, two screening phases were carried out to filter publications. In the first screening phase, we filtered publications based on whether they are reviews, or their titles and abstracts are relevant, and then 44 papers were retained. In the second screening phase, as the scope of our review focuses on the research area of NLP applications in oncology, we filtered papers based on whether their topics fit our scope or the NLP model is relevant to computer-aided diagnosis, and then 23 papers were retained. The process of searching is illustrated in Figure 3. For more visible, the inclusion criteria and exclusion criteria of the articles are shown in Table 2.

## 4. Results

**Figure 4:**
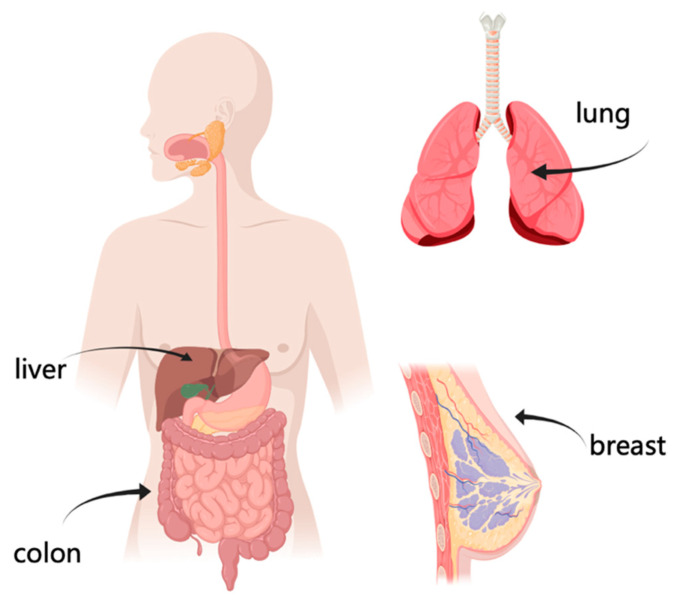
The human organ anatomy diagram includes breast, colon, lung, and liver.

To summarize the application of NLP for CAD in cancer and tumors, we conducted an analysis based on the content of selected publications. We presented the studies in this review in terms of the year, text source, cancer type, purpose, algorithm, evaluation metrics, and dataset. Diagnosis is a classification in computer tasks, so all words like “prediction”, “identification”, “classification”, or the noun form of these words in the original text were replaced with “classification” or “classification” in the purpose column. For ease of viewing, we arranged the studies of the same tumor type in the same table in descending order by year. Table 3, Table 4, Table 5 and Table 6 show studies on seven types of tumors: breast cancer, lung cancer, liver cancer, prostate cancer, pancreatic cancer, colorectal cancer, and brain tumors. In the Evaluation Metrics column, we recorded the performance of the best-performing models in the paper. In addition, we provided an explanation of models that have not been introduced before in the overview of each paper. Figure 4 illustrates different human organs such as breast, colon, lung, and liver. In this section, we categorize 23 included studies into four subsections: breast cancer, colorectal cancer, lung cancer, and other cancers (liver cancer, prostate cancer, pancreatic cancer, and brain tumors).

### 4.1. Breast Cancer

Breast cancer is the most commonly diagnosed female cancer and has a leading mortality rate of patients with cancer in women [14]. Hence, to investigate how NLP can assist clinicians in diagnosing breast cancer, we identify 12 studies about the NLP applications on breast cancer, and we analyze each study from multiple perspectives. BERT [15] used a masked language model (MLM) to pretrain bidirectional Transformers to generate deep bidirectional language representations. Kaka et al. [16] used BERT models with a consistent network structure but based on different datasets: BERT-base (general text dataset) and ClinicalBioBERT (biomedical text and clinical text dataset) [17] to predict the recurrence of colorectal and breast cancers, respectively. By comparing the experimental results in internal datasets of BERT-base and ClinicalBioBERT, the authors found that they differ by one standard deviation, indicating that the BERT only needs a certain size of dataset sufficient to learn the features of cancer recurrence without specialized knowledge. Deshmukh et al. [18] used a rule-based algorithm to extract clinical factors and applied them to an ML model to predict the anatomic stage and prognostic stage. In terms of model selection, because of the performance and interpretability of decision trees (DT), the authors chose decision trees over Gaussian Naive Bayes (GNB) and linear support vector machine (SVM). GNB refers to the assumption that the conditional probabilities of each feature dimension of a sample obey Gaussian distribution, then the model calculates the posterior probability of a new sample belonging to each category under a certain feature distribution according to the Bayesian formula, and finally determines the category of the sample by maximizing the posterior probability. This study used datasets from two different institutions to improve the generalizability of the prognostic system compared to those from past studies.

Sanyal et al. [19] developed a weakly supervised framework for breast cancer recurrence prediction using LSTM to simulate labeling on the original unlabeled dataset. The experimental results confirmed that training with the generated dataset gave better results than training with only manually labeled data. The datasets used by Sanyal et al. are large compared to other studies, so the experimental results are also relatively more reliable. In [20], to build interpretable neural networks, the authors first embed semantic trees into BERT and used a capsule network to improve the semantic representation of multiple heads of attention. Then, backpropagation and dynamic routing algorithms enable the local interpretability of the model. This study presents the first model combining capsule networks with semantic embedding for breast tumor diagnosis. Alzu’bi et al. [21] extracted key features of breast cancer from EMR with the features integrated to construct a dictionary of breast cancer. The authors tested multiple machine learning algorithms to predict breast cancer recurrence based on this dictionary. The OneR algorithm had the best performance balance. The core of OneR is to find the most important feature among all the features of the dataset for classification. The experimental results were approved by professional doctors, proving that the prediction can help them to make the right decision on specific treatment options.

Wang et al. [22] transformed clinical notes into concept unified identifiers (CUI), which are fed into a variant model of CNN, Knowledge-Guided Convolutional Neural Network (K-CNN) [23], to predict the distant recurrent probability of breast cancer. The authors experimented with the different features as inputs, and finally the experiments yielded an AUC of 0.888 and an f1-score of 0.5. This research work was dedicated to models that require less specialized knowledge and data collation than the previous studies to predict the distant recurrence of breast cancer. In [24], a neural network-based NLP system was developed to determine the timeline for patient-specific recurrence of metastatic breast cancer. The authors compared the model with a rule-based algorithm and found its sensitivity to be superior to the rule-based algorithm. In conclusion, the authors proposed a new strategy to exploit the predictive potential of EMR-based data on metastatic cancer recurrence. Zeng et al. [25] used MetaMap, a rule-based software, to extract positive features in sentences indicating local recurrence of breast cancer and developed an SVM model to identify local recurrence of breast cancer. The authors obtained the best AUC by comparing the model with three baseline models: using the full MetaMap concept, the filtered MetaMap concept, or the word package. In [26], Breitenstein et al. constructed the rule-based NLP algorithm from both the prescribing and clinical narrative perspectives, which can derive breast cancer receptor status phenotypes in both structured and unstructured EHR data. This study took an informatics approach to propose that NLP can provide annotations for the specific clinical data elements.

Bozkurt et al. [27] developed an NLP system to predict the degree of malignancy of a lesion. The input is the extraction of BI-RADS descriptors and clinical information from X-ray reports, and the body of the algorithm is a Bayesian network (a probabilistic graphical model). The output is the probability of malignancy and the category of Bi-RADS assessment. The model’s accuracy in predicting the Bi-RADS final assessment category was 97.58%, sufficient to provide accurate decision results, as assessed experimentally. This was the first study to assess the impact of imperfections in automated information extraction on model accuracy. In [28], the authors developed rules to extract the parameters: tumor (T), lymph node (N), and metastasis (M) to determine the T, N, and stage of breast cancer. The accuracy of the model for cancer staging was obtained up to 72%. Carrell [29] designed the rule-based NLP system using cTAKEs software to determine whether and when breast cancer recurrence is diagnosed in EHR. The system is based on the pathology module, an element describing breast cancer, and the clinical module, a positive reference to breast cancer recurrence in the report. With an accuracy of 92% and a sensitivity of 96%, the system can replace human annotation of EHR to a certain extent for reducing labor costs.

**Table 3:** Breast cancer-related studies. * represents that we only show the size of the dataset because the dataset has too many types of data (more than three), the distribution of the dataset is not fully described, or it is not easy to show the structure of the dataset for other reasons.

SN	Reference	Year	Source of Text	Language	Cancer Type	Aim	Algorithm	Evaluation Metrics	Validation	Dataset Size	Dataset Source
1	[16]	2022	Medical Notes (Unstructured)	English	Breast Cancer, Colorectal Cancer	Classify Cancer Recurrence	Bidirectional Encoder Representations from Transformers (BERT) [15]	Breast Cancer: AUC: 0.9892; Colorectal Cancer: AUC: 0.9810;	5-fold Cross-validation	Breast Cancer: 190,754 Notes; 8067 Positive; 182,687 Negative Colorectal Cancer: 238,408 Notes; 8452 Positive; 229,956 Negative	Private: From Cancer Care Manitoba
2	[18]	2021	Medical Records (Unstructured)	English	Breast Cancer	Classify Breast Cancer Anatomic and Prognostic Stage	Decision Tree	Anatomic: Rural Accuracy: 0.93; Urban Accuracy 0.86; Rural F1-score 0.9638; Urban F1-score 0.9123; Prognostic: Rural Accuracy: 0.92; Urban Accuracy: 0.82; Rural F1-score: 0.9521; Urban F1-score: 0.8765;	5-fold Cross-validation	465 Medical Records *	Private: From India’s cancer treatment institutions (Nurgis Dutta Memorial Cancer Hospital in the rural region and Jehangir Hospital urban and laboratories in the urban region)
3	[19]	2021	Free-text Clinical Notes (Unstructured)	English	Breast Cancer	Classify Breast Cancer Recurrence	Long Short-Term Memory (LSTM)	AUC 0.94; Sensitivity 0.89; Specificity 0.84;	5-fold Cross-validation	Embedding: 92.6 million Clinical Notes Prediction: 892,550 Clinical Notes *	Public: Clinical language space: I2B2 NLP research database [30], MIMIC-III critical care database [31], Oncoshare breast cancer database [32]
4	[20]	2021	Mammography Reports (Unstructured)	Chinese	Breast Cancer	Classify Breast Cancer	BERT	Micro: AUC: 0.94; Precision: 0.9158; Recall: 0.9158; F1-score: 0.9158; Macro: AUC: 0.85; Precision: 0.7595; Recall: 0.7973; F1-score: 0.7714	N/A	2857 Mammography Reports; 2078 Benign; 448 Suspected of Malignant; 331 Malignant	Private: From Shanghai Ruijin Hospital
5	[21]	2021	Histopathology Report (Unstructured)	English	Breast Cancer	Classify Breast Cancer Recurrence	One Rule (OneR)	Accuracy: 0.901; Sensitivity: 0.901; Specificity: 0.722;	10-fold Cross-validation	142 Histopathology Report *	Private: From King Abdullah University Hospital (KAUH) in Jordan
6	[22]	2020	Progress Notes and Pathology Notes of EHR (Unstructured + Structured)	English	Breast Cancer	Classify Breast Cancer Recurrence	Knowledge-guided Convolutional Neural Networks (K-CNN)	AUC: 0.888; Precision: 0.537; Recall: 0.468; F1-score: 0.500; Specificity: 0.968;	5-fold Cross-validation	6447 Subjects; 446 Positive; 6001 Negative	Private: From Northwestern Medicine Enterprise Data Warehouse (NMEDW)
7	[24]	2019	Clinical Notes (Unstructured)	English	Breast Cancer	Classify Breast Cancer Recurrence	Neural Network	Quarter-Level: AUC 0.9; Definite Recurrence: Specificity 0.82; Sensitivity 0.73; F1-score 0.77; No Recurrence: Specificity 0.99; Sensitivity 0.99; F1-score 0.99; Patient-Level: Specificity 0.95; Sensitivity 0.93; F1-score 0.94;	Validation	894 Subjects *	Public: Oncoshare breast cancer database [32]
8	[25]	2018	Pathology Reports of EHR (Unstructured)	English	Breast Cancer	Classify Breast Cancer Recurrence	Support Vector Machine (SVM)	Precision 0.5; Recall 0.81; F1-score: 0.62; AUC: 0.87;	5-fold Cross-validation	6899 Subjects; 581 Positive; 6318 Negative;	Private: From Northwestern Medicine Enterprise Data Warehouse (NMEDW).
9	[26]	2018	EHR (Unstructured + Structured)	English	Breast Cancer	Classify Derived Breast Cancer (BC) Receptor Status Phenotypes	Rule-based	Estrogen Receptor (ER): Precision: 0.9758; Recall: 0.9877; F1-score: 0.9818; Progesterone Receptor (PR): Precision: 0.9857; Recall: 0.9418; F1-score: 0.9632; Human Epidermal Growth Factor Receptor 2 (HER2): Precision: 0.6977; Recall: 0.6667; F1-score: 0.6818; Triple Negative (TN): Precision: 0.7222; Recall: 0.6848; F1-score: 0.7027	N/A	871 Subjects *	Private: From Mayo Clinic, Rochester, Minnesota
10	[27]	2016	Mammography Reports (Unstructured)	English	Breast Cancer	Classify Breast Cancer	Bayesian Network (BN)	Accuracy 0.9815;	N/A	300 Mammography Reports *	Private: From An Academic Radiology Practice
11	[28]	2015	Pathology reports (Unstructured)	English	Breast Cancer	Classify the Breast Cancer Stages	Rule-based	Tumor (T) Classification: Precision: 0.79; Recall: 0.75; Accuracy: 0.76, Lymph Nodes (N) Classification: Precision: 0.81; Recall: 0.63; Accuracy: 0.66; Cancer Stage Classification: Precision: 0.729; Recall: 0.825; Specificity: 0.587; NPV: 0.711; Accuracy: 0.722	N/A	150 Pathology Reports *	Private: From Christian Medical College and Hospital
12	[29]	2014	Clinical Text of EHR (Unstructured)	English	Breast Cancer	Classify Breast Cancer Recurrence	Clinical Text Analysis and Knowledge Extraction System (cTAKES)	Sensitivity: 0.92; Specificity: 0.96; PPV: 0.66; F1-score: 0.76;	N/A	1472 Subjects; 141 Positive; 1331 Negative	Private: From the Commonly Used Medications and Breast Cancer Recurrence (COMBO) Study Conducted at Group Health, An Integrated Health Care Delivery System in the Pacific Northwest

### 4.2. Colorectal Cancer

Regarding cancer mortality rates, colorectal cancer is the third leading cause of death for both men and women. Approximately 1.85 million cases of colorectal cancer occur worldwide every year, resulting in 850,000 deaths [33]. In this review, we identify four studies on colorectal cancer-related NLP applications. Cheng [34] applied CNN to identify primary colon cancer in cases and achieved an accuracy of 92%. This work demonstrates the high accuracy of CNN in the problem of making dichotomous classifications of cases. Parthasarathy et al. [35] developed an NLP method using the Prolog language that can identify serrated polyposis syndrome (SPS) from EMR. The method follows the rules based on the WHO criteria. Raju et al. [36] developed a rule-based NLP method to detect adenomas and sessile serrated adenomas (SSAs) in first-screening colonoscopy. The experiments show that the accuracy of this NLP method is higher than that of the manual method. The authors developed such a complete and practical diagnostic system for colonoscopy in 2015, which is enlightening for subsequent studies. Similarly, in [37], the authors designed a rule-based system using KMCI to identify colorectal cancer test and patients who need screening. To ensure the performance of the model, the authors expanded the synonyms of the relevant concepts as much as possible when developing the rules. The NLP system was also used in the study to count whether the CRC test was accepted by patients, which is a valuable indicator for doctors to assess CRC screening.

**Table 4:** Colorectal cancer-related studies. * represents that we only show the size of the dataset because the dataset has too many types of data (more than three), the distribution of the dataset is not fully described, or it is not easy to show the structure of the dataset for other reasons.

SN	Reference	Year	Source of Text	Language	Cancer Type	Aim	Algorithm	Evaluation Metrics	Validation	Dataset Size	Dataset Source
1	[34]	2022	Pathology Reports (Unstructured)	English	Colorectal Cancer	Classify Cases with Primary Colonic Adenocarcinoma	CNN	Accuracy: 0.92; AUC 0.957	Validation	1000 Anatomic Pathology Reports; 713 Positive; 287 Negative	N/A
2	[35]	2020	Colonoscopy and Pathology Reports of EMR (Unstructured)	English	Colorectal Cancer	Classify Serrated Polyposis Syndrome (SPS)	Rule-based	Accuracy: 0.93	N/A	255,074 Patients; 71 Positive; 255,003 Negative	Private: From Cleveland Clinic, Cleveland, Ohio
3	[36]	2015	Pathology and Colonoscopy Reports (Unstructured)	English	Colorectal Cancer	Classify Adenomas and Sessile Serrated Adenomas (SSAs)	Rule-based	Screening Accuracy: 0.913; Adenomas Accuracy: 0.994; SSAs Accuracy: 1;	N/A	12,748 Patients; 2288 Positive; 10,460 Negative	Private: From the University of Texas MD Anderson Cancer Center
4	[37]	2012	EHR (Unstructured + Structured)	English	Colorectal Cancer	Classify the Colorectal Cancer (CRC) Test, Classify Patients in Need of Screening	Knowledge Map Concept Identifier (KMCI)	CRC Classification: Recall: 0.93; Precision: 0.94; F1-score: 0.94; Patients Classification: Recall: 0.95; Precision: 0.88; F1-score: 0.91;	N/A	500 EHR Records *	Private: From four Vanderbilt University Medical Center (VUMC)-affiliated ambulatory health care clinics in Nashville, Tennessee

### 4.3. Lung Cancer

In the United States, lung cancer is one of the most common malignant tumors, and the second most commonly diagnosed cancer ranked after prostate cancer in men and breast cancer in women [38]. We analyzed three papers on applying NLP to lung cancer-related computer-aided diagnosis. Nobel [39] used a rule-based algorithm to classify the radiologically reported T-stage of pulmonary tumors. The algorithm was developed based on Dutch with rules translated into English and applied to an English dataset. The possibility of applying rule-based algorithms to multiple languages was demonstrated. In addition, the authors designed a graphical user interface to visualize the algorithm. Yuan et al. [40] modeled the classification of lung cancer and prognostic survival of non-small cell lung cancer based on logistic regression and cox regression. Cox regression is a semi-parametric regression model. By comparing the results of the trials, the authors confirmed that lung cancer classification and prognostic survival prediction models could be used to enhance the entire cohort of EHR. The entire EHR cohort can be continued for other prognostic studies. Wadia et al. [41] used cTAKES to investigate a rule-based algorithm to distinguish benign lesions from lung cancer. The experimental results show that the model is even more sensitive than manual screening. The innovation of this study is that it is the first to compare NLP and manual coding with reference standards established by clinicians in unselected radiology reports.

**Table 5:** Lung cancer-related studies. * represents that we only show the size of the dataset because the dataset has too many types of data (more than three), the distribution of the dataset is not fully described, or it is not easy to show the structure of the dataset for other reasons.

SN	Reference	Year	Source of Text	Language	Cancer Type	Aim	Algorithm	Evaluation Metrics	Validation	Dataset Size	Dataset Source
1	[39]	2021	Free-text Radiological Reports (Unstructured)	English	Lung Cancer	Classify T-stage and T-substage	Rule-based	T-stage: Accuracy: 0.89; T-substage: Accuracy: 0.84; Average Precision: 0.8375; Average Recall: 0.825; Average F1-score: 0.81375;	N/A	425 Radiological Reports *	Private: From the Departments of Radiation Oncology and Radiology, Brigham and Women’s Hospital/Dana-Farber Cancer Institute (Boston, United States of America)
2	[40]	2021	EHR (Unstructured + Structured)	English	Lung Cancer	Classify Lung Cancer and Prognostic	Lung Cancer Classification: Logistic Regression, Prognostic Classification: Cox Regression	Lung Cancer: AUC: 0.927; Specificity: 0.9; Sensitivity: 0.752; Precision: 0.994; F1-score: 0.837; Prognostic: AUC (1-year): 0.828; AUC (2-year): 0.825; AUC (3-year): 0.814; AUC (4-year): 0.814; AUC (5-year): 0.812;	Cross-validation	76,643 Patients *	Private: From Massachusetts General Hospital (MGH) and Brigham and Women’s Hospital
3	[41]	2018	CT Reports (Unstructured)	English	Lung Cancer	Classify Lung Cancer	cTAKES	Sensitivity: 0.773; Specificity: 0.725; PPV: 0.884; NPV: 0.54;	N/A	446 Chest CT Reports; 326 Positive; 120 Negative	Private: From Veterans Affairs Connecticut Healthcare System

### 4.4. Other Cancers

Besides the common cancers, such as breast, colorectal, and lung cancer, we also collect and analyze four studies on other cancer types, including brain tumor, liver, prostate, and pancreatic cancer. In [42], Liu et al. applied BiLSTM, which is a variant of LSTM that collects sequence information in both directions to the NER task for identifying features in EMR, which is effective because the NER task is essentially a sequence labeling task. Furthermore, the authors applied different machine learning methods to construct a classification model for liver cancer prediction, and random forest [43], which is a classification model containing many decision trees, had the highest performance in this task. This study focused on the limited previous studies of Chinese radiology reports that have significant implications for the research on NLP. In [44], Lee et al. developed an NLP model for the automatic classification of brain tumors. For structured and unstructured MR reports, the authors applied different feature engineering: Tf-idf and word2vec. An ensemble of ElasticNet [45], which is a regression model, random forest, and XGBoost [46], which is a frame to ensemble models, was used in the backbone of the machine learning algorithm. Bozkurt et al. [47] separately designed a rule-based NLP algorithm and a CNN algorithm to classify UI. They found that the results of the rule-based NLP algorithm were better than those of the CNN algorithm and slightly better than the combination of the rule-based NLP algorithm and CNN. Roch et al. [48] implemented an NLP system to identify pancreatic cysts automatically. The algorithm defined regular expressions for nine different pancreatic cysts using rules and incorporated negation detection in specific sentence screening.

**Table 6:** Liver cancer, brain tumor, pancreatic cancer, and prostate cancer-related studies. * represents that we only show the size of the dataset because the dataset has too many types of data (more than three), the distribution of the dataset is not fully described, or it is not easy to show the structure of the dataset for other reasons.

SN	Reference	Year	Source of Text	Language	Cancer Type	Aim	Algorithm	Evaluation Metrics	Validation	Dataset Size	Dataset Source
1	[42]	2020	Radiology Reports of EMR (Unstructured)	Chinese	Liver Cancer	Named Entity Recognition (NER), Classify Liver Cancer	NER: Bidirectional Long Short-term Memory (BiLSTM), Liver Cancer Classification: Random Forest	NER: Precision: 0.9235; Recall: 0.9366; F1-score: 0.9300; Liver Cancer Classification: Precision: 0.8771; Recall: 0.8625; F1-score: 0.8697	5-fold Cross-validation	609 Radiology Reports *	Private: From Beijing Friendship Hospital, Capital Medical University, Beijing, China
2	[44]	2020	Magnetic Resonance Imaging (MR) Reports (Unstructured + Structured)	English	Brain Tumor	Classify Brain Tumor	Ensemble Model (ElasticNet + RandomForest + Gradient boosting (XGBoost))	Structured Text (Tf-idf + Ensemble): F1-score: 0.98; Unstructured Text (word2vec + Ensemble): 0.72;	N/A	26,000 Brain MR Reports; 1410 BT-RADS Reports *	Private: From a Single Academic Institution
3	[47]	2020	Clinical Notes of EHR (Unstructured)	English	Prostate Cancer	Classify Urinary Incontinence (UI)	Rule-based	Accuracy 0.86; Average Precision: 0.957; Average Recall: 0.833; Average F1-score: 0.887;	5-fold Cross-validation for CNN	259 Clinical Notes; 87 Mild; 79 Moderate; 93 Severe	Private: From the Stanford University EHR with the Stanford Cancer Institute Research Database (SCIRDB) and the California Cancer Registry (CCR)
4	[48]	2015	Free Text of EMR (Unstructured)	English	Pancreatic Cancer	Classify Pancreatic cyst	Rule-based	Mean Sensitivity: 0.9985; Mean Specificity: 0.988;	N/A	566,233 Reports *	Private: From Wishard Memorial Hospital

## 5. Discussion

The recent advances in AI and DL have revolutionized the field of medicine, including computer-aided diagnosis and radiology [49], while most of these studies have only applied DL-enabled computer vision (CV) algorithms. However, how DL-enabled NLP as well as the conventional NLP techniques can be utilized in computer-aided diagnosis has not been well-investigated. In the era of COVID-19, medical resources are significantly important and doctors are always busy. We find that AI-enabled NLP techniques still have not been applied in hospitals. Many activities, such as medical reports writing, clinical notes analysis, and EMR analysis, still take up a large amount of time for doctors. By using advanced NLP methods to build applications, we believe that these applications can improve the efficiency of doctors. In this section, we provide the answers to the three RQs that we provided in the Introduction Section. Based on the results and the analysis of this review, we answer RQ1 in Section 5.1. In addition, we answer RQ2 and RQ3 in Section 5.2 and Section 5.3, respectively.

### 5.1. Current Trends

In this section, we summarize the current trends of the NLP applications for CAD in oncology from two perspectives: (1) NLP algorithms and (2) datasets and disease types.

#### 5.1.1. NLP Algorithms

**Figure 5:**
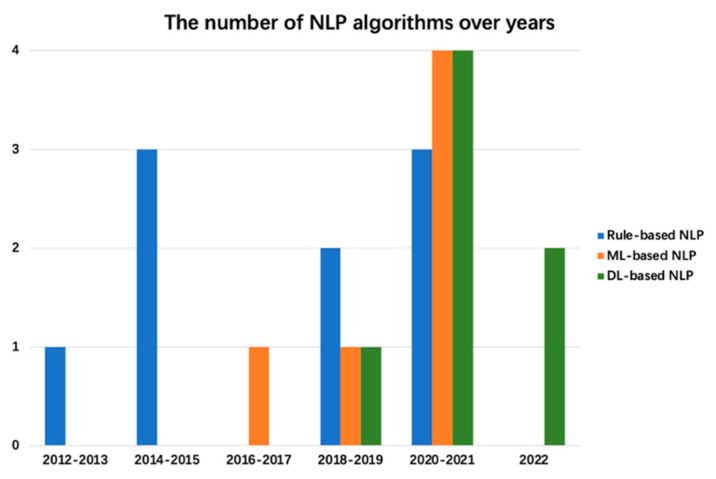
The number of NLP algorithms over the years (2012–2022): a comparison of rule-based NLP algorithms, ML-based NLP algorithms, and DL-based NLP algorithms.

Figure 5 shows the number of different NLP algorithms for CAD in oncology from 2012 to 2022. Until 2020, the mainstream algorithm model was the rule-based NLP algorithm. Between 2016 and 2019, ML-based NLP models and DL-based NLP models were only sequentially applied in this field. Since 2020, the share of rule-based NLP algorithms has been gradually decreasing, while ML-based NLP algorithms and DL-based NLP algorithms have been gradually increasing in use and becoming the mainstream methods. In general, the development of NLP applications in this field can basically match the iteration of AI technology power. However, to a certain extent, it also exposes the gap between the models used in the field and the frontier models of NLP. For example, in the last three years, researchers have still been using rule-based NLP algorithms.

In the last three years, NLP models applied in oncology for CAD still have rule-based algorithms. Retro models are still in use today, while no one uses more advanced NLP models than BERT, proving that the models applied in oncology are somewhat cut off from the advanced NLP models. In today’s widespread use of pre-trained models, we can develop state-of-the-art NLP models using the Hugging Face library [50] at a low cost. Moreover, we can apply some advanced training methods to the models for practical application implications. For example, we can replace the normal dropout layer [51] with a multiple-sample dropout layer [52] to improve the generalization ability of the model. Alternatively, we can insert a simple LSTM model at the end of the complex model as a pooling layer. Moreover, in some cases, we apply the pre-trained model directly to specific downstream problems. Its performance is much higher than the rule-based algorithm. Of course, such a technical divide arises not only because of the information gap between the NLP domain and the oncology domain. Physicians may also not fully trust the diagnoses predicted by AI models and are reluctant to do the relevant research. Rule-based models, in which every rule is developed by human experts, have a proven track record of diagnostic results. AI models are black-box models; thus, it is difficult to interpret the right and wrong diagnoses for samples. Such uninterpretability is extremely detrimental to the development of AI models in CAD. 

#### 5.1.2. Datasets and Disease Types

**Figure 6:**
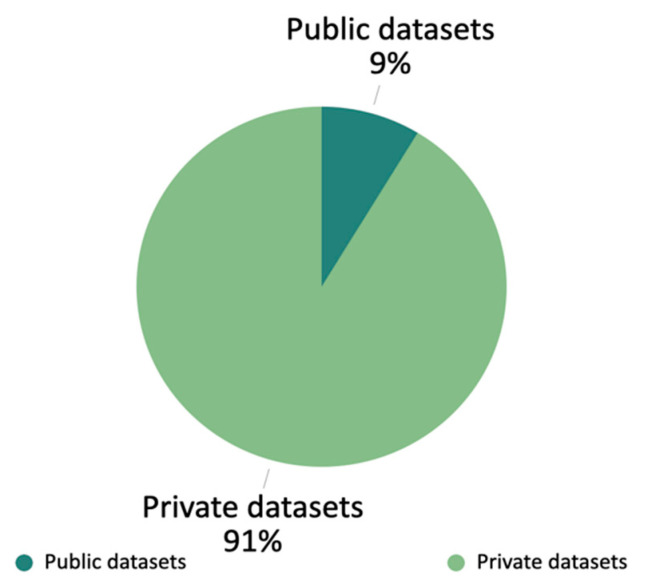
The ratio of the number of the papers using datasets: a comparison of public datasets to private datasets.

We identified three public datasets in the field of NLP applications in oncology: the I2B2 NLP research database [30], the MIMIC-III critical care database [31], and the Oncoshare breast cancer database [32]. The rest of the datasets are private datasets obtained by the authors in collaboration with local medical institutions. Figure 6 shows the ratio of the number of papers using private datasets to the number of the papers using public datasets. The percentage of public datasets in the studies we count is low. This is due to patient privacy and data security issues, which make data collection challenging to carry out. In addition, the annotations of electronic medical records are costly. For different annotations, it is often necessary to be develop special annotation tools and refer to experienced doctors. The Chinese-based dataset studies account for 2 out of 23 statistical articles, indicating that NLP in oncology based on the English datasets is the mainstream study. From the tables in Section 4, we observe that the sources of text for all the studies include unstructured data because unstructured data contain more clinical information than structured data for the models to make predictions. Moreover, it is the role of NLP to convert unstructured data into structured results on demand. In terms of dataset size, the tables in Section 4 show that some datasets are much smaller than those for other non-medical NLP tasks, such as sentiment analysis. Furthermore, the classes in some datasets are severely uneven. More discussion of the datasets is in Section 5.2.1.

**Figure 7:**
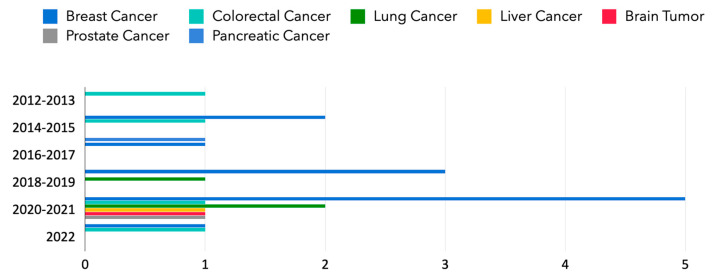
The evolution of different types of cancers over the years (2012–2022): a comparison of breast cancer, colorectal cancer, lung cancer, liver cancer, brain tumor, prostate cancer, and pancreatic cancer.

In terms of the disease types, among the studies, breast cancer has been most extensively studied (12/23), followed by colorectal cancer (4/23) and lung cancer (3/23). However, only a few studies worked on brain tumors (1/23), pancreatic cancer (1/23), prostate cancer (1/23), and liver cancer (1/23). Figure 7 shows the evolution of different types of cancers over the years. In [53], the authors estimated that the most common cancer in 2040 in the United States would be breast cancer, with 364,000 cases. Moreover, lung cancer was estimated to be the leading cause for cancers, with 63,000 deaths. In this survey, 52.17% of included papers are breast cancer-related, which corresponds to the fact that breast cancer is the most common type. With the increasing estimated diagnosed cancer patients and cancer-related death, the NLP applications may provide help and serve as an important part in the future.

### 5.2. Challenges

The challenges in this area can be summarized into two main limitations: the limitations of the dataset and the limitations of the validation methods.

#### 5.2.1. Dataset Limitations

A limiting factor noted by Deshmukh and Phalnikar [18] in doing the prognostic stages of breast cancer is the insufficient number of datasets resulting in unbalanced datasets. The unbalanced distribution of datasets leads to a classification with a small sample size containing too few features, and it is not easy to extract patterns from them. Classification models are prone to the problem of over-fitting due to over-dependence on a limited data sample. When the model is applied to new data, the accuracy and robustness of the model will be poor. In [16,34], KaKa et al. and Cheng proposed that the dataset is all from the same institution and that the data homogeneity will impact the generalization ability of the model. In addition, Cheng [34] also mentioned that the predictions of DL-based NLP are based on matrix calculations where all features are converted into numbers, reducing the interpretability of the algorithm. Alzu’bi [21] et al. mentioned that the quality of the collected data itself might have some influence on the whole experimental process, such as the variable format of data and missing data.

In summary, the limitations of datasets can be divided into insufficient original datasets and inadequate quality original datasets. The data drive the NLP model kernel. The dataset affects the model’s performance, and such an impact may even be in the tens of percentage points. The performance expectations of models in the medical field are demanding. So, if we want the models in our study to be truly useful, we should filter the valid datasets. Additionally, the small size dataset in this field may be highly challenging to develop DL-based models. Attributed to the data-driven approach, a small size dataset may cause over-fitting, and the model may not be robust to the new unseen data. More research should be focused on addressing this challenge.

#### 5.2.2. Validation Limitations

The validation of model performance is what we generally want to test to the most extent is the model’s generalization ability. The generalization ability means the true metrics of the model in dealing with real-world data. However, we found some problems in the validation steps of NLP models within the CAD for oncology. The most common problem involves not using cross-validation. The partial dataset used for NLP modeling in oncology CAD has only a few hundred cases. This can lead to differences between individual samples when we randomly divide the training and validation sets, resulting in unstable model training results due to randomness. Nevertheless, if we combine multiple models generated by cross-validation, the performance of the models will be more stable, and the final validation results will be more reliable. Furthermore, we found that, in some studies, only one expert manually annotated the dataset, or only one round of annotation was performed. This corresponds to many subjective opinions in the final validation results, resulting in a lack of objectivity. We propose to eliminate subjective differences by combining the annotation results of multiple professional researchers and performing multiple rounds of cross-annotation.

### 5.3. Future Trends

#### 5.3.1. Federated Learning

Text data in the field of CAD for oncology are special because of the need to consider patient privacy. It was mentioned earlier that we need to consider acquiring more datasets if we want NLP to continue in the field of CAD for oncology; however, it is difficult to consider the data security perspective if we are to collaborate with multiple medical centers to share data for this purpose. Federated learning (FL) was proposed by McMahan et al. [54], which is represented a distributed approach to training ML models without requiring private data. FL exists in the form of a client and a corresponding server. The parameters are shared among the various untrusted clients and finally aggregated into a federated model. There are already current applications of FL in the context of the medical field. For example, FL is used for whole-brain segmentation [55] and brain tumor segmentation in MRI [56]. Basu et al. [57] investigated some effects of applying Differential Privacy (DP) in FL on BERT-like models. This study can be used to protect medical history against privacy in the future.

#### 5.3.2. Explainable Artificial Intelligence

DL-based black box models have no way to make sense of the decisions involved. Therefore, people cannot trust their predictive abilities and do not know when they will fail to predict. Such shortcomings prevent people from completely deploying them to some critical areas that require performance, such as oncology. In addition to this, the need for interpretability or explainable artificial intelligence (XAI) is higher in medicine than in other fields. The reasons are that we need to identify the risks and liabilities in the medical process [58] and unexplained clinical diagnoses can undermine trust between patients and doctors [59]. Nurdin and Adi [60] parse two different models of deep learning using three models of interpretation. The behavior of these two models on a sentiment analysis task was investigated. Trigueros et al. [61] used a CNN with an attention mechanism to detect which part of the EHR led to the output, generating interpretable predictions. There are not yet many medical studies dealing with interpretability. However, from some existing examples, interpretability can facilitate other studies to obtain more conclusive model information. This feature is needed for NLP models in the field of CAD for oncology.

#### 5.3.3. Semi-Supervised Learning

The data within the field of oncology belong to the medical field, so they inherit the special characteristics of medical data. In terms of dataset size, medical data will be much smaller than the data in other fields. Medical data often require tagging by multiple doctors, and such tagging is too expensive. Semi-supervised learning can be well integrated into this domain in such a data context. Semi-supervised learning allows us to acquire a small amount of labeled data and a large amount of unlabeled data to train models and improve model performance. Yang et al. [62] combined semi-supervised learning with generative adversarial networks (GAN) to support clinical decision making. Liu et al. [63] proposed semi-supervised learning for extracting clinical features of Traditional Chinese Medicine and applied semi-supervised learning based on BiLSTM (Bidirectional LSTM)-CRF to balance the cost of manual annotation and model performance.

## 6. Conclusions

Electronic health records and electronic medical records are collected in hospitals, but doctors have not utilized these data efficiently. Recently, natural language processing techniques have been applied in the EHR and EMR data and play an essential role in the clinical environment to assist doctors in cancer diagnosis. However, to the best of our knowledge, no review paper has summarized the NLP applications for computer-aided diagnosis in oncology. To fill the gap, we conducted a literature review from PubMed, ACL Anthology, and Google Scholar between 2012 and 2022, and we finally included 23 papers. Moreover, we analyzed and categorized the articles into seven cancer types: breast cancer, lung cancer, liver cancer, prostate cancer, pancreatic cancer, colorectal cancer, and brain tumors. We have found that DL-based and ML-based approaches have been more widely used recently, while rule-based approaches were the dominant solution in earlier years. Additionally, by analyzing the literature, we identified the current limitations of NLP applications on supporting clinical practices and suggested some promising future research directions. The current challenges are the limitation of the dataset size, lack of usage of cross-validation, and lack of standard validation mechanisms. Some promising future trends, such as federated learning, explainable artificial intelligence, and semi-supervised learning, should be investigated to address these challenges. We believe this multidisciplinary survey may reduce the gap between AI experts and medical professionals and provide the necessary support for future researchers to design NLP applications for CAD in oncology.

## Figures and Tables

**Table 1:** The details of evaluation metrics used for NLP models.

Method	Formula	Description
Accuracy	$\frac{\left(\mathrm{TP}+\mathrm{TN}\right)}{\mathrm{TP}+\mathrm{FP}+\mathrm{TN}+\mathrm{FN}}$	Percentage of total sample with correct predictions
Precision	$\frac{\mathrm{TP}}{\mathrm{TP}+\mathrm{FP}}$	The probability of all samples predicted to be positive being truly positive
Recall/Sensitivity/TPR	$\frac{\mathrm{TP}}{\mathrm{TP}+\mathrm{FN}}$	The probability of samples that are truly positive being predicted as positive samples
Specificity/PPV	$\frac{\mathrm{TN}}{\mathrm{TN}+\mathrm{FP}}$	The probability of samples that are truly negative being predicted as negative samples
NPV	$\frac{\mathrm{TN}}{\mathrm{TN}+\mathrm{FN}}$	The probability that following a negative test result, that samples will truly be negative
FPR	$\frac{\mathrm{FP}}{\mathrm{FP}+\mathrm{TN}}$	The probability between the number of negative samples incorrectly classified as positive and the total number of actual negative samples
F-score/F1	$\frac{2\times \mathrm{Precision}\times \mathrm{Recall}}{\mathrm{Precision}+\mathrm{Recall}}$	The maximum balance between recall and precision of the model
ROC	N/A	A more comprehensive evaluation of the model using the curves constructed from sensitivity and specificity
AUC	N/A	Area under the ROC curve

Abbreviations: FN = False Negative, FP = False Positive, TN = True Negative, TP = True Positive, TPR = True Positive Rate, FPR = False Positive Rate, PPV = Positive Predictive Value, NPV = Negative Predictive Value.

**Table 2:** The inclusion criteria and exclusion criteria of the articles.

First Screening	Second Screening
To screen out the reviews (exclusion criterion)	To filter out whether the purposes in full text are consistent with diagnosis (inclusion criterion)
To remove the duplicate papers (exclusion criterion)	To choose the papers whose specific role of NLP models in full text is the computer-aided diagnosis (inclusion criterion)
To filter out the papers based on their titles and abstracts (exclusion criterion)	

## Data Availability

Not applicable.
